# Peer Review of “No Time to Waste: Real-World Repurposing of Generic Drugs as a Multifaceted Strategy Against COVID-19”

**DOI:** 10.2196/24453

**Published:** 2020-09-30

**Authors:** Ahmed Abdeen Hamed

**Affiliations:** 1 School of Cybersecurity, Data Science and Computing Norwich University Northfield, VT United States

**Keywords:** COVID-19, drug repurposing

*This is a peer review submitted for the paper “No Time to Waste: Real-World Repurposing of Generic Drugs as a Multifaceted Strategy Against COVID-19.”*

## Round 1 Review

### General Comments

The title of the manuscript presents the urgent topic of drug repurposing in the context of COVID-19. Clearly, research in this area is much needed, and the esteemed authors have put it well: “no time to lose.” The manuscript, however, suffers from some significant and obvious issues that can be summarized as follows:

### Specific Comments

#### Major Comments

1. The esteemed authors didn’t use the preferred template. Particularly, the abstract section, if the template is followed, should summarize the research background, objectives, methods, results, and conclusions. These sections are not clear from the current abstract.
2. The manuscript is missing significant sections such as the Methods section, which is the most exciting part of any paper. It is not clear how the esteemed authors have summarized the literature (methods, experiments, tools, development environment, etc). The only methods that were mentioned was in reference number 67, but the paper has entirely missed this very significant section (among many others).
3. The manuscript is also missing significant content that fails to demonstrate how the analysis was conducted and how the results are presented. The manuscript does not have a single figure and has only one table.
4. The esteemed authors have immediately presented their classes (which I believe to be the Conclusion section) after the Background section. Since it does not follow the JMIR template, this manuscript does not make it possible for the information in this paper to be accessible to the reader.
5. The esteemed authors have presented a report or an early stage whitepaper that can potentially be turned into a research paper. However, in the current stage, I don’t believe it qualifies as a publishable manuscript (not without adding a section on methods, experiments, etc, and following the recommended template). I highly recommend to the esteemed authors to search the JMIR archives for keywords such as literature, COVID, and drug repurposing and find some publications that they can potentially use as a guide to present their work.

